# Supplementary material for: Opportunistic Premise Plumbing Pathogens. A Potential Health Risk in Water Mist Systems Used as a Cooling Intervention
Source: Pathogens. 2021 Apr 12;10(4):462. doi: 10.3390/pathogens10040462 (PMC8068904; doi:10.3390/pathogens10040462)
Supplement: Supplementary file 1 [file pathogens-10-00462-s001.zip › Supplementary files/PCR and qPCR primer sequencies and cycling conditions used during the study.docx]

PCR/qPCR Primer sequencies and cycling conditions used during the study

| ***Pathogen*** | **Cycling conditions** | **PCR/qPCR Primer sequencies** |
| --- | --- | --- |
| *Legionella spp.* | 1. Initial denaturation cycle of 1 minute at 95 °C 2. 30 cycles for denaturation at 95 °C for 5s  3. 30 cycles of annealing at 60 °C for 10 s  4. Extension at 72 °C for 15s  5. End holding cycle for 7 minutes at 72 °C PCR fragments 79 bp (10 % tolerance), 110 pb (10% tolerance) and 124 (5% tolerance) | ***PCR Collins et al., 2015*** *Legsp-F (5’ – NGG CGA CCT GGC TTC -3’); Legsp-R (5’- GGT CAT CGT TTG CAT TTA TAT TTA – 3’) Lp-mip-F2 (5’ – TTG TCT TAT AGC ATT GGT GCC G – 3’) Lp-mip-R (5’ – CCA ATT GAG CGC CAC TCA TAG – 3’) Lp-wzm-F(5’ – TGC CTC TGG CTT AGC AGT TA – 3’) Lp-wzm-R(5’ – CAC ACA GGC ACA GCA GAA ACA -3’)* |
| *P. aeruginosa* | *1. 1 denaturation cycle at 95 °C for 3 min 2. 35 cycles with each one made up of: - 1m at 94 °C, 68 °C for 90s, 72 °C for 1 min 3. Extension cycle of 10 min at 72 °C* | ***qPCR Khan & Cerniglia, 1994***  *F: (ETA1: 5’-GAC AAC GCC CTC AGC ATC ACC AGC-3’) R: (ETA2: 5’-CGC TGG CCC ATT CGC TCC AGC GCT-3’)* |
| *M. avium* | *1. 1 denaturation cycle at 95 °C for 8 minutes*  2. *29 amplification cycles made up of: 3. Denaturation for 60s at 95 °C*  4. A*nnealing for 60s at 40 °C*  *5. Extension for 35s at 72 °C 6. Last extension cycle for 10 minutes at 72 °C* | ***qPCR Uppal et. al., 2002***  *F: (5’ATAAGCCTGGGAAACTGGGT3’) R:(5’CACGCTCACAGTTAAGCCGT3’) F:(5’ GCGTGAGGCTCTGTGGTGAA3’) R:(5’ATGACGACCGCTTGGGAGAC3’)* |
| *Acanthamoeba* | *1. 1 cycle for initial denaturation at 95 °C for 5min 2. 40 cycles with each made up of: - denaturation at 95 °C for 30s annealing at 56 °C for 30s extension at 72 °C for 1min 3. 1 cycle for holding at 72 °C for 7 min* | ***PCR Schroeder et al., 2001*** *JDPI F: (5’-GGCCCAGATCGTTTACCGTGAA) JDP2 R:(5’-TCTCACAAGCTGCTAGGGAGTCA)* |
| *N. fowelri* | *1. 1 cycle for initial activation at 95 °C for 5 min 2. 60 cycles for denaturation at 95 °C for 10s 3. 60 cycles of annealing and extension at 95 °C for 45s* | ***PCR Puzon et al., 2009***  *F: (5’-GAA CCT GCG TAG GGA TCA TTT) R:(5’ -TTT CTT TTC CTC CCC TTA TTA -3’) F:(5’ GTG AAA ACC TTT TTT CCA TTT -3’) R:(5’ TTT CTT TTC CTC CCC TTA TTA -3’)* |
