# Supplementary material for: Opportunistic Premise Plumbing Pathogens. A Potential Health Risk in Water Mist Systems Used as a Cooling Intervention
Source: Pathogens. 2021 Apr 12;10(4):462. doi: 10.3390/pathogens10040462 (PMC8068904; doi:10.3390/pathogens10040462)
Supplement: Supplementary file 1 [file pathogens-10-00462-s001.zip › Supplementary files/Summary of analytical methods used during the study.docx]

Summary of analytical methods used during the study

|  |  | **Sample Matrix** | | |
| --- | --- | --- | --- | --- |
| **OPPP** |  | **Bioaerosol** | **Biofilm** | **Water** |
| *Legionella* spp. | *Analytical Method/Sensitivity*/LOR*** | ***PCR*** *[80****] Sensitivity = 1.6 Genomic Units/mL*** | ***EDP-312****(Culture = PCR)*  *Culture followed by confirmation by PCR Culture LOR = 10 CFU/mL* | ***EDP-312*** *(Culture + PCR)*  *Culture followed by confirmation by PCR Culture LOR = 10 CFU/mL* |
| *Pseudomonas aeruginosa* | *Analytical Method/Sensitivity*/LOR*** | ***qPCR*** *[82]*  *Sensitivity = 5-10 GU/10mL* | *AS 4276.13(****EDP-306****)*  *Culture followed by confirmation by qPCR Culture LOR = 1 CFU/100mL* | *AS 4276.13(****EDP-306****)*  *Culture followed by confirmation by qPCR Culture LOR = 1 CFU/100mL* |
| *Mycobacterium avium* | *Analytical Method/Sensitivity*/LOR*** | ***qPCR*** *[86] Sensitivity = 5 - 10 GU/*µ*L* | *qPCR Uppal et. al., 2003* | *qPCR Uppal et. al., 2004* |
| *Acanthamoeba* | *Analytical Method/Sensitivity*/LOR*** | ***PCR*** *[85]*  *Sensitivity = 5 - 8 gene copies/*µL | ***EDP-315*** *(Culture + PCR)*  *Culture followed by confirmation by PCR Culture LOR = 3.5 CFU/250mL* | ***EDP-315*** *(Culture + PCR)*  *Culture followed by confirmation by PCR Culture LOR = 3.5 CFU/250mL* |
| *Naegleria fowleri* | *Analytical Method/Sensitivity*/LOR*** | ***PCR*** *[83,84] Sensitivity = 12 - 17 gene copies/*µ*L* | ***EDP-314*** *(Culture +PCR)*  *Culture followed by confirmation by Culture LOR = 3.5 CFU/250mL* | ***EDP-314*** *(Culture +PCR)*  *Culture followed by confirmation by Culture LOR = 3.5 CFU/250mL* |

**= LOR -*Limit of Reporting (Expressed as such because of the need to do serial dilutions before performing test): *= PCR/qPCR Sensitivity*

*Methods prefixed with “****EDP****” are covered under the laboratory’s Australian National Association of Testing Authorities (NATA) Accreditation Number: 19290*

***References***

*80. Collins S, Jorgensen F, Willis C, Walker J. Real-time PCR to supplement gold-standard culture-based detection of Legionella in environmental samples. J Appl Microbiol. 2015;119(4):1158-69.*

*82. Khan AA, Cerniglia CE. Detection of Pseudomonas aeruginosa from clinical and environmental samples by amplification of the exotoxin a gene using PCR. Appl Environ Microbiol. 1994;60(10):3739(7).*

1. *Pélandakis, M.; Serre, S.; Pernin, P. Analysis of the 5.8 S rRNA gene and the internal transcribed spacers in Naegleria spp. and in N. fowleri. J. Eukaryot. Microbiol.* ***2000****, 47, 116–121.*

*84. Puzon GJ, Lancaster JA, Wylie JT, Plumb JJ. Rapid Detection of Naegleria Fowleri in Water Distribution Pipeline Biofilms and Drinking Water Samples. Environ Sci Technol. 2009;43(17):6691.*

*85. Schroeder JM, Booton GC, Hay J, Niszl IA, Seal DV, Markus MB, et al. Use of subgenic 18S ribosomal DNA PCR and sequencing for genus and genotype identification of Acanthamoebae from humans with keratitis and from sewage sludge. J Clin Microbiol. 2001;39(5):1903-11.*

*86. Uppal M, McLellan S, Collins M, Lambrecht R, editors. A multiplex PCR assay that discriminates Mycobacterium avium subspecies paratuberculosis from closely related Mycobacteria using primers which detect specific insertion sequences, abstr. Z-58. Abstr 102nd Annu Meet Am Soc Microbiol American Society for Microbiology, Washington, DC; 2002.*
